# Fracture Strength of Three-Unit Implant Supported Fixed Partial Dentures with Excessive Crown Height Fabricated from Different Materials

**Published:** 2016-11

**Authors:** Vahideh Nazari, Safoura Ghodsi, Marzieh Alikhasi, Majid Sahebi, Ahmad Reza Shamshiri

**Affiliations:** 1 Assistant Professor, Department of Prosthodontics, Faculty of Dentistry, Arak University of Medical Sciences, Arak, Iran; 2 Assistant Professor, Dental Research Center, Dentistry Research Institute, Tehran University of Medical Sciences, Tehran, Iran; Department of Prosthodontics, School of Dentistry, Tehran University of Medical Sciences, Tehran, Iran; 3 Associate Professor, Dental Research Center, Dentistry Research Institute, Tehran University of Medical Sciences, Tehran, Iran; Department of Prosthodontics, School of Dentistry, Tehran University of Medical Sciences, Tehran, Iran; 4 Assistant Professor, Research Center for Caries Prevention, Dentistry Research Institute, Tehran University of Medical Sciences, Tehran, Iran; Department of Community Oral Health, School of Dentistry, Tehran University of Medical Sciences, Tehran, Iran

**Keywords:** Dental Implants, Polyetheretherketone, Zirconium Oxide, Dental Restoration Failure, Dental Porcelain

## Abstract

**Objectives:**

Fracture strength is an important factor influencing the clinical long-term success of implant-supported prostheses especially in high stress situations like excessive crown height space (CHS). The purpose of this study was to compare the fracture strength of implant-supported fixed partial dentures (FPDs) with excessive crown height, fabricated from three different materials.

**Materials and Methods:**

Two implants with corresponding abutments were mounted in a metal model that simulated mandibular second premolar and second molar. Thirty 3-unit frameworks with supportive anatomical design were fabricated using zirconia, nickel-chromium alloy (Ni-Cr), and polyetheretherketone (PEEK) (n=10). After veneering, the CHS was equal to 15mm. Then; samples were axially loaded on the center of pontics until fracture in a universal testing machine at a crosshead speed of 0.5 mm/minute. The failure load data were analyzed by one-way ANOVA and Games-Howell tests at significance level of 0.05.

**Results:**

The mean failure loads for zirconia, Ni-Cr and PEEK restorations were 2086±362N, 5591±1200N and 1430±262N, respectively. There were significant differences in the mean failure loads of the three groups (P<0.001). The fracture modes in zirconia, metal ceramic and PEEK restorations were cohesive, mixed and adhesive type, respectively.

**Conclusions:**

According to the findings of this study, all implant supported three-unit FPDs fabricated of zirconia, metal ceramic and PEEK materials are capable to withstand bite force (even para-functions) in the molar region with excessive CHS.

## INTRODUCTION

With development of dental implants, prosthetic treatment for replacement of missing teeth has significantly improved [1]. However, selection of prosthetic materials is a critical factor determining the long-term clinical success and stability of implant prostheses. Prosthetic material influences the transmission mechanism of stress created during function; this stress can be transferred to the prosthetic components, implant and bone-implant interface [1–3]. This is particularly important in high stress situations like excessive crown height space (CHS) that can technically influence clinical outcomes [4]. It has been shown that by increasing the CHS, resistance to loading decreases significantly [5]. Metal-ceramic fixed partial dentures (FPDs) have long been used and are the gold standard fixed dental prostheses [6,7]. Base metal alloys such as nickel-chromium (Ni-Cr) are preferred over noble alloys for implant supported metal ceramic restorations due to lower cost and higher elastic moduli, hardness, fracture strength [8], and high porcelain to metal bond strength [9]. More recently, yttria-stabilized tetragonal zirconia polycrystalline (Y-TZP) zirconia frameworks were developed as an esthetic alternative for metal ceramic implant restorations [10] due to high chemical, mechanical, physical and optical properties, and good clinical success even in the posterior region [11].

Polyetheretherketone (PEEK) is a high-performance engineering plastic, which has attracted attentions of dental researchers [12,13]. Low modulus of elasticity (4 Gpa) of PEEK results in its shock-absorbing effect that makes it a suitable material for implant prostheses in load-bearing areas such as excessive CHS [14]. However, there are limited data available on fracture strength of PEEK restorations.

As mentioned before, the load bearing capacity of implant prostheses depends on the material’s properties. However, few articles have addressed the failure loads of metal-ceramic and PEEK restorations, and there is no study on fracture strength of implant prostheses fabricated with excessive CHS. The purpose of this in-vitro study was to examine the fracture strength of posterior implant supported three-unit FPDs with excessive CHS fabricated from zirconia, Ni-Cr, and PEEK after veneering. The null hypothesis was that the choice of material would have no significant effect on fracture strength of implant supported FPDs.

## MATERIALS AND METHODS

**Fig. 1:**
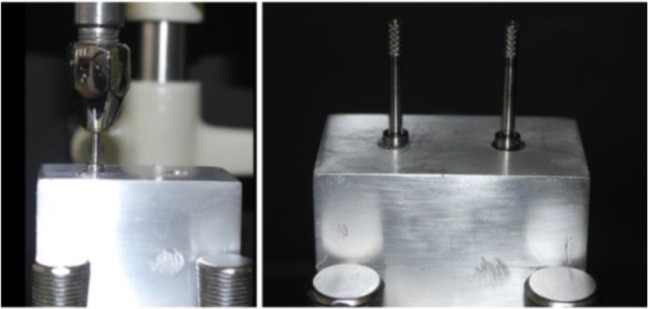
Mounted implants parallel to each other using a surveyor

Two implants, 12mm in height and 4.5mm in diameter (Implantium; Dentium, Seoul, South Korea), were inserted in an aluminum reference model using auto-polymerizing acrylic resin (Technovits 4000; Heraeus Kulzer GmbH & Co., Wehrheim, Germany). The distance between the centers of the implants was 18mm. This distance was approximately equal to the distance between the centers of the mandibular second premolar and the second molar. A dental surveyor (J.M. Ney Co., Bloomfield, CT) was used to control the vertical and axial orientation of inserted implants (Fig. 1). Two straight abutments (Implantium; Dentium, Seoul, South Korea) with 5.5mm height and 4.5 and 5.5mm platform diameter were adapted over each implant and tightened to 35Ncm according to the manufacturer’s instructions. All samples were directly fabricated on this reference model.

### Framework fabrication:

Three groups of mandibular posterior three-unit FPDs were constructed. Each group consisted of 10 samples. The control group was manufactured from Ni-Cr alloy (4all; Ivoclar Vivadent, Schaan, Liechtenstein) and two other groups were manufactured from Y-TZP (VITA In-ceram YZ; VITA Zahnfabrik, Bad Säckingen, Germany) and PEEK (Bio-HPP; Bredent GmbH &Co.KG, Senden, Germany) using computer aided design/computer aided manufacturing system.

For fabrication of PEEK and zirconia frameworks, the reference model was sprayed (VITA Powder Scan Spray; VITA Zahnfabrik, Bad Säckingen, Germany) and scanned (3shapeD810; 3Shape, Copenhagen, Denmark). The frameworks were designed using the 3Shape Dental System software (3Shape, Copenhagen, Denmark). The substructures were designed in supported anatomical form with a 3mm collar height in lingual and proximal surfaces. The crown height was set at 15mm and the thickness of the veneering was considered 1.5mm in occlusal and 0.8mm in axial surfaces. Data were subsequently transferred to the computer aided manufacturing unit (CORiTEC450i; Imes-icore GmbH, Eiterfeld, Germany), where the presintered VITA In-Ceram YZ and PEEK blocks were milled.

The milled zirconia substructures were heated to a completely sintered state in a high temperature furnace (VITA ZYRCOMA; Vita Zahnfabrik, Bad Säckingen, Germany).

For fabrication of Ni-Cr frameworks, the abutments were coated with die spacer (PICOFIT red; Renfert GmbH, Hilzingen, Germany), and wax patterns were formed using inlay wax (GEO classic; Renfert GmbH, Hilzingen, Germany) based on an index fabricated from the first zirconia framework.

The samples were cast by Ni-Cr alloy using a casting machine (Nautilus CC Plus; Bego, Bremen, Germany). The castings were blasted with 50μm aluminum-oxide particles at a pressure of 0.3MPa.

### Veneering process:

To standardize the ceramic layer contour on all frameworks, one of the zirconia frameworks was veneered (VITA VM9; Zahnfabrik, Bad Säckingen, Germany), and the second silicone index was fabricated. Afterwards, the Ni-Cr substructures were veneered with VITA VMK Master (Zahnfabrik, Bad Säckingen, Germany). After blasting the PEEK frameworks with 110μm aluminum-oxide particles at 2.5 bar pressure, a light-cure adhesive (Visio.link; Bredent GmbH & Co KG, Senden, Germany) was applied and light cured for 90 seconds. Then, the PEEK frameworks were veneered with composite (Crea.lign; Bredent GmbH & Co KG, Senden, Germany).

### Assessment of fracture strength:

**Fig. 2:**
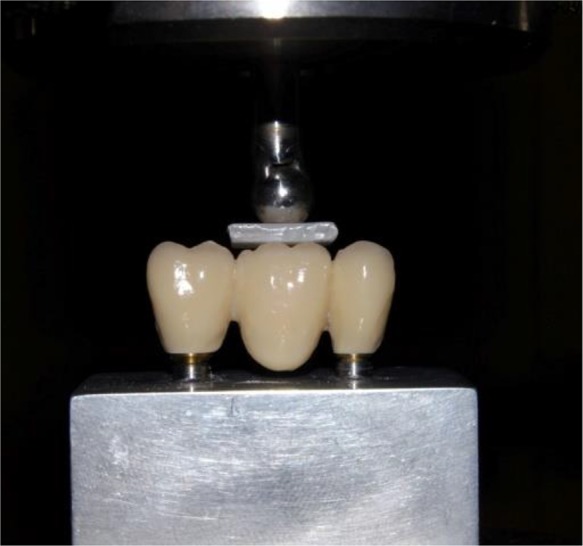
Position of stainless steel ball and Teflon disk at loading point

Each framework was placed on the abutments and then the assembly was mounted on a universal testing machine (Z050; Zwick Roell, Ulm, Germany). Axial compressive load was applied to the center of the occlusal surface of pontic using a stainless steel ball (diameter = 8mm) at a constant crosshead speed of 0.5 mm/minute. To prevent any contact damage and to provide homogeneous load distribution on the pontic, a 2mm thick Teflon disk with length and width of 10×10mm, was placed between the stainless steel ball and the occlusal surface of the pontic (Fig. 2). Loading was continued until catastrophic fracture of the samples occurred. The data of failure load were recorded using computer software (Zwick's test Xpert v2; Zwick, Ulm, Germany), and registered in Newtons (N).

Additionally, the location and nature of the fracture mode were recorded using a stereomicroscope (Leitz GmbH & Co. KG, Oberkochen, Germany) at ×50 magnification and a camera (5MP Edge AM7115MZT; Dino-Lite, Naarden, Netherlands). Fracture mode was divided into three patterns namely adhesive (fracture at the interface of framework and veneering), cohesive (fracture in one of the substrates i.e. framework or veneering) and mixed (combination of adhesive and cohesive fractures).

### Statistical analysis:

The fracture strength values of all samples were reported as mean and standard deviation (SD) and were analyzed statistically using one-way ANOVA followed by Games-Howell test for pairwise comparisons. The level of significance was considered as P< 0.05.

## RESULTS

The mean (±SD) fracture loads of zirconia, Ni-Cr and PEEK groups were 2086.31±362.61, 5591.74±1200.29 and 1430.47±262.21N, respectively. The fracture load of the Ni-Cr group was significantly higher than that of the zirconia and PEEK groups (P<0.001). The zirconia restorations had significantly higher fracture strength than the PEEK restorations (P<0.001).

The fracture mode in all metal-ceramic samples was mixed (combination of adhesive and cohesive in the veneering porcelain) and Ni-Cr framework remained intact. The fractures occurred in lingual surface of pontic in three cases, and in buccal surface of pontic in seven cases. In all the zirconia samples, fractures were oblique and located between loading point and one of the connectors. Six samples fractured through the distal (molar) connector and four samples fractured through the mesial (premolar) connector. The fracture mode in all zirconia samples was cohesive, which initially occurred in the veneering porcelain and was then propagated to the framework.

**Fig. 3:**
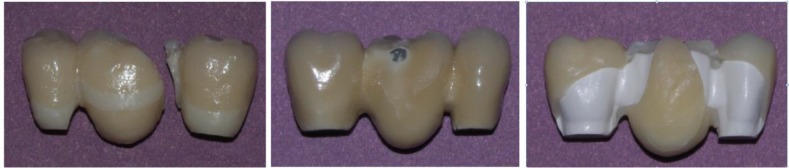
Examples of a fractured FPD (left to right: zirconia, metal ceramic and PEEK restorations)

In four samples, adhesive failure was also observed between the framework and porcelain. The fracture mode of all PEEK samples was adhesive, which occurred between the framework and the veneering composite. In four samples, the failure was confined to the pontic and in six samples, the fracture extended to the molar and premolar retainers (Fig. 3).

## DISCUSSION

Fracture strength of prosthetic materials is important to predict the clinical service and failure rates. This experimental study evaluated the fracture strength of posterior three-unit implant-supported FPDs with excessive CHS fabricated from zirconia, PEEK, and Ni-Cr after veneering. The results support the rejection of the null hypothesis because significant differences were observed.

In review of the literature, there were limited articles on the fracture load of metal ceramic implant supported FPDs [7]. In the present study, fracture load of metal ceramic restorations was calculated to be 5591N. In an in vitro study conducted by Chitmongkolduk et al [15], the fracture load of 3500N was reported for three-unit metal ceramic FPDs, with almost similar dimensions to the FPDs in our study.

Deviations in the reported fracture loads in these two studies may be explained by different abutment materials, alloy type, cementation procedures or bridge designs. In our study, fractures observed in metal ceramic restorations were mixed (combination of cohesive failure in the veneering porcelain and adhesive failure between the veneering porcelain and Ni-Cr framework) and were mostly located in the buccal surface of pontics. It seems that existence of lingual collar helps to strength the veneering porcelain in the lingual surface.

Fracture strength of metal ceramic bridge was significantly higher than that of other groups. The mean value of fracture strength in zirconia prostheses was 2086N. This is significantly higher than the maximum bite force reported in the molar region in cases with parafunctional habits such as bruxism, which ranges from 500 N to 880N [16,17].

In several in vitro studies, the mean fracture load of Y-TZP based all-ceramic FPDs is reported to be in the range of 386N to 3480N [6,11,18,19]. Different experimental designs and testing parameters, such as abutment material and mobility, veneering, cementation, shape and size of connectors, inclination of applied load, and fatigue loading were used in previous in vitro studies; this makes it rather difficult to compare the results of previous studies with ours [7,20]. In our study, the fracture pattern of all zirconia samples was oblique, occurring in the loading point and extending through one connector. This type of fracture pattern has also been observed in previous studies [20–23].

It was difficult to determine the initiation point of fractures whether to be at the connector or at the loading point on the pontic. Finite element analysis and photoelastic studies have shown that during axial compressive loading of the pontic, gingival embrasures of both connectors (beside pontic) are subjected to the highest tensile stress while the occlusal surface of both connectors (beside pontic) and the abutment side of the gingival connector, as well as the loading point on the pontic, are subjected to the highest compressive stress [18,20– 22].

Since the tensile stress produced during loading is tolerated poorly compared to compressive stress in ceramic materials, it was assumed that cracks start from the gingival embrasure of one connector and spread toward the loading point at the center of occlusal surface of the pontic [18,23]. In our study, fractures developed almost equally in the mesial and distal connectors. Fracture strength of PEEK restorations was significantly lower than that of metal ceramic and zirconia groups. No study has evaluated the fracture strength of PEEK restorations after composite veneering so far.

The mean fracture strength of the PEEK restorations in the current study was 1430N, which was considerably lower than the mean fracture load of the two other groups, but considerably higher than that of the reported physiological maximum posterior masticatory force of 880N [16,17]. Therefore, PEEK restorations fabricated in excessive CHS can potentially withstand physiological occlusal forces.

In this study, the failure mode of all PEEK restorations was adhesive between the frameworks and the veneering composite. This failure mode is the result of the weak bond strength of PEEK frameworks to composite resin owing to hydrophobic, chemically inert surface of PEEK, and its resistance to surface modification by different chemical treatments [12,13,24–27].

Only one study investigated the fracture strength of PEEK three-unit FDPs before veneering and showed a mean fracture load of 1383N [28]. As stated earlier, the design of FPD influences the fracture strength. In the afore-mentioned study, the PEEK frameworks had connectors with dimensions of 3.2×2.3mm and uniform design without lingual shoulder [28]; this factor is probably responsible for framework fracture at a lower force compared to the results of our study. In the present study, tested samples were not cemented on the abutments, and were not exposed to fatigue loading, which could be regarded as the study limitations. Further studies with better simulation of oral conditions are required to investigate the fracture strength of implant restorations under dynamic forces of mastication or fatigue loading, because static axial loading cannot always simulate actual functional conditions.

## CONCLUSION

Within the limitations of this in vitro study, it can be concluded that implant supported three-unit FPDs fabricated from zirconia, metal ceramic, and PEEK materials in excessive CHS have the potential to withstand occlusal forces. Metal ceramic restorations have higher fracture strength than PEEK or Zirconia restorations to withstand occlusal forces.
